# Relationship Between Developmental Dislocation of the Hip in Infant and Acetabular Dysplasia at Skeletal Maturity

**DOI:** 10.1097/MD.0000000000000268

**Published:** 2015-01-09

**Authors:** Kunihiko Okano, Kazumasa Yamaguchi, Yoshikazu Ninomiya, Shohei Matsubayashi, Kiyoshi Aoyagi, Makoto Osaki, Hiroshi Enomoto, Katsuro Takahashi

**Affiliations:** Department of Orthopaedic Surgery, Nagasaki Prefectural Center of Medicine and Welfare for Children, Isahaya, Japan (KO, KY, YN, SM); Department of Public Health, Nagasaki University Graduate School of Biomedical Sciences, Nagasaki, Japan (KA); Department of Orthopedic Surgery, Graduate School of Biomedical Sciences, Nagasaki University, Nagasaki, Japan (MO); Enomoto Orthopaedic Clinic, Nagasaki, Japan (HE); and Takahashi Orthopedic Clinic, Nagasaki, Japan (KT).

## Abstract

Previous reports demonstrated 8–60% patients treated for developmental dislocation of hip (DDH) in infancy have residual acetabular dysplasia (AD) at skeletal maturity. AD patients reportedly exhibit abnormal morphology of the pelvis, high rates of comorbid spinal congenital anomalies and high bone mineral density. These physical findings suggest that AD patients have genetic background. We examined the percentage of AD patients with hip pain at skeletal maturity having a history of DDH in infancy and the correlation between the severity of AD at skeletal maturity and history of DDH treatment to investigate the relationship between AD and DDH.

A total of 245 patients were radiographically examined for any history of DDH treatment in infancy. The study included 226 women and 19 men with a mean age at examination of 40.7 years (range 17–59 years).

Eighty-eight patients (36%) had a history of DDH treatment (DDH group) and the remaining 157 patients (64%) had no history of DDH treatment (non-DDH group). The average age was lower and acetabular angle was larger in the DDH group. There was a significant increasing trend of the percentage of DDH patients associated with the severity of AD classified with CE, acetabular angle, and acetabular roof angle.

Our data suggest that there are several AD patients without a history of DDH in Japan, and AD in patients without a history of DDH has different characteristics from AD in patients with a history of DDH.

## INTRODUCTION

Developmental dysplasia of the hip comprises of disorders of hip development that present in different forms at different ages. The common etiology is excessive laxity of the hip capsule, which fails to maintain the femoral head within the acetabulum. In newborns, the syndrome consists of instability of the hip such that the femoral head can be displaced from the acetabulum. The hip may also rest in a dislocated position and be reducible. Over time, the femoral head becomes fully dislocated and cannot be reduced by changing the position of the hip. The syndrome may manifest later in childhood as a developmental dislocation of the hip (DDH) or in adolescence as a hip with poorly developed acetabular coverage; the latter is termed acetabular dysplasia (AD).^1^

In general, infants are screened for DDH in the first 4 months of life by clinical examination. If the infant demonstrates limited abduction of the affected hip, further physical examination, plain radiography, and an ultrasound are performed. An experienced orthopedic surgeon confirms DDH diagnosis. Infants diagnosed with DDH are usually treated with a Pavlik harness at the time of diagnosis. In addition, traction, closed reduction, and open reduction procedures are also selected as treatment options depending on the patient's age.^1^ From 8% to 60% of patients with a history of treatment for DDH have been reported to have residual AD at skeletal maturity.^2–4^ Osteoarthritis (OA) is an age-related degenerative disease that is common in both middle-aged and older women.^5^ Primary OA of the hip is an extremely rare condition in Japan; most patients have secondary OA due to AD and DDH.^6^ Japanese patients with OA of the hip have been reported to have higher rates of dysplasia than American patients (46% vs 4.5%),^7^ and significantly worse dysplasia compared with that in British populations (mean center-edge angle^8^ of 37° vs 31°).^9^

Patients with AD have been reported to exhibit an abnormal morphology of the pelvis^10,11^ and have a high rate of comorbid spinal congenital anomalies, such as spina bifida occulta.^12^ In a recent study, bone mineral density was reported to be higher in patients with AD than that in normal controls.^13^ However, the precise pathomechanism of AD remains unknown. These reports suggest there is a genetic link to the development of AD.

In this study, to investigate the relationship between AD and DDH, we examined the percentage of AD patients with hip pain at skeletal maturity also having a history of DDH in infancy and the correlation between the severity of AD at skeletal maturity and a history of treatment for DDH.

## MATERIALS AND METHODS

This retrospective diagnostic study received permission for publication from the institutional review board of our institute.

For this study, we selected patients diagnosed with AD of the hip as well as pre-arthritis or early-stage osteoarthritis. Patients with advanced or end-stage osteoarthritis were excluded from the study so that we could more accurately evaluate AD by excluding osteophyte formation.^14^ Patients less than 16 years of age were not included to exclude premature hip joints and those older than 60 years were excluded because of the difficulty of confirming a history of treatment for DDH. In addition, patients with a history of hip osteotomy, hip dislocation into the gluteal muscles, secondary post-traumatic osteoarthritis, inflammatory rheumatic disease, osteonecrosis, or infectious diseases were excluded from the study.

All patients in the study visited our hospital for consultation regarding hip joint pain between 2009 and 2012. A total of 245 patients were radiographically examined and questioned for any history of DDH treatment in infancy. The study population comprised 226 women and 19 men with a mean age at examination of 40.7 years (range 17–59 years).

**FIGURE 1:**
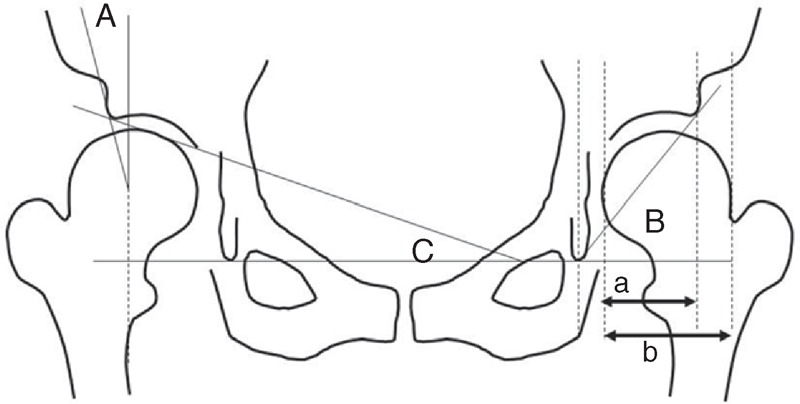
Diagram showing the radiological parameters (A, center-edge angle; B, acetabular angle; C, acetabular roof angle; acetabular head index (AHI) = *a*/*b* × 100).

Study parameters evaluated were center-edge angle (CE angle),^8^ acetabular head index (AHI),^15^ acetabular angle^16^, and acetabular roof angle^17^ (Figure 1). AD was defined as a CE angle less than 20°, AHI less than 75%, acetabular angle more than 45°, or acetabular roof angle more than 15° on anteroposterior radiographs.^18^ When bilateral AD was observed in a patient, the joint with a higher degree of AD was chosen for analysis.

Severity of osteoarthritis was graded using the Japanese Orthopedic Association criteria.^19^ Osteoarthritis of the hip was classified into the following four stages: pre-arthritis stage with no osteoarthritic change; early stage with narrowing of the joint space associated with sclerosis of the subchondral bone; advanced stage with partial disappearance of the joint space and evidence of cystic radiolucencies and osteophytes, and end stage with almost a total disappearance of the joint space and marked osteophyte formation.

All radiographs were performed in the supine position. Anteroposterior radiographs were taken using a source-to-film distance of 110 cm. The patient's feet were internally rotated with the toes at 15° ± 5° to ensure that the X-ray beam was centered on the superior aspect of the pubic symphysis.

To analyze the relationship between the severity of AD at skeletal maturity and history of treatment for DDH, patients were divided into three groups (mild, moderate, and severe AD), comprising equal number of patients (Table 1).

**TABLE 1:**
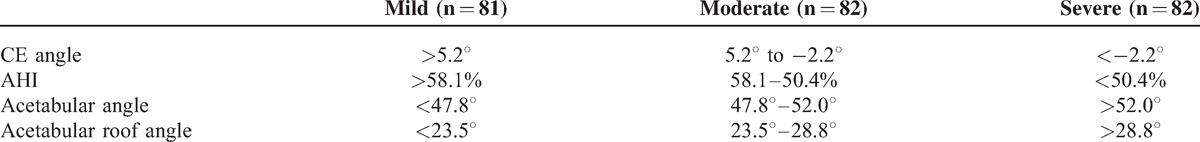
Variables in Mild, Moderate, and Severe Acetabular Dysplasia Groups

To test the reproducibility of the radiographic measurements, three authors (KO, KY, and YN) measured the CE angle, AHI, acetabular angle, and acetabular roof angle in five randomly selected hips. Each hip was measured three times with an interval of 1 week between measurements, and the values were subsequently averaged. The data were analyzed for intra- and inter-observer variances, and the coefficient of variation was calculated to be less than 5%. Therefore, the reproducibility of the measurements was considered reasonable.

Differences between values in DDH and non-DDH groups were tested using the Mann–Whitney *U* test. Differences in the percentage of DDH patients between the mild and severe dysplasia groups were tested using Fisher exact test. The Cochran–Armitage test was used to test for trends in the percentage of DDH patients according to the three groups. Logistic regression analysis was used to evaluate the simultaneous effect of various factors on DDH. The significance level of the hypothesis test was chosen as *P* < 0.05. All statistical analyses were performed with EZR (Saitama Medical Center, Jichi Medical University).

## RESULTS

A total of 88 patients (36%) had a past history of treatment for DDH (DDH group) and the remaining 157 patients (64%) had no history of DDH (non-DDH group). Although the average age was lower and the acetabular angle was larger in DDH group, no significant differences of height, weight, CE angle, AHI, and acetabular roof angle were observed between the DDH and non-DDH groups (Table 2). Younger age was significantly associated with DDH after adjustment for CE angle, AHI, acetabular angle, and acetabular roof angle [Odds ratio (OR) = 1.09, 95% confidence interval (CI) = 1.06–1.13, *P* < 0.0001].

**TABLE 2:**
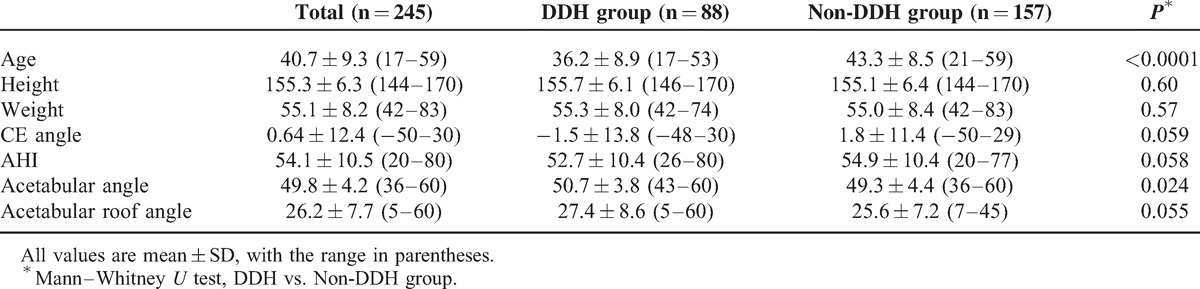
Variables in Total, DDH, and Non-DDH Group

**FIGURE 2:**
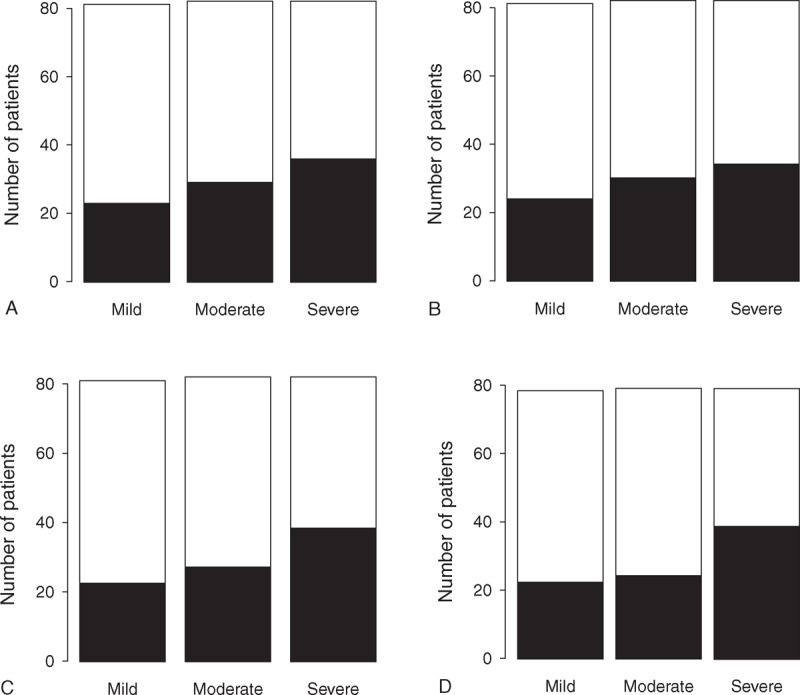
Graph showing the number of DDH (black square) and non-DDH (white square) patients in three groups (mild, moderate, and severe dysplasia) classified by CE angle (Figure 2A), AHI (Figure 2B), acetabular angle (Figure 2C), and acetabular roof angle (Figure 2D).

There were significant differences in percentages of DDH and non-DDH patients between the mild and severe dysplasia groups classified by acetabular angle (*P* = 0.048, Figure 2C) and acetabular roof angle (*P* = 0.013, Figure 2D); however, no differences were observed on the basis of CE (*P* = 0.12) (Figure 2A) and AHI (*P* = 0.28) (Figure 2B). There were significantly increasing trends in the percentage of DDH patients according to severity of AD classified on the basis of CE (*P* = 0.039) (Figure 2A), acetabular angle (*P* = 0.017) (Figure 2C), and acetabular roof angle (*P* = 0.0066) (Figure 2D). However, no trend was observed among the three groups classified on the basis of AHI (*P* = 0.12) (Figure 2B).

## DISCUSSION

Previous reports have described that 8 to 60% patients treated for DDH in infancy have residual AD at skeletal maturity.^2–4^ In this study, we investigated the relationship between DDH in infancy and the later development of AD. We observed that 64% patients with AD at skeletal maturity have no history of treatment for DDH. Although there was an increasing trend in the percentage of DDH patients associated with severity of AD at skeletal maturity, more than half of the patients with severe AD at skeletal maturity had no history of treatment for DDH.

Our study had several limitations with regard to obtaining the treatment history of DDH patients. First, with regard to questioning patients and their parents on the type of treatment received for DDH during infancy, although we were able to obtain treatment history from all patients in the DDH group, we were not able to confirm certain details, such as the side of the dislocated hip or the exact treatment method employed. These patients could describe the treatment history, but were unable to provide detailed aspects of the treatment methods used. Second, there is a possibility that the non-DDH group in the present study may have included patients with untreated DDH that spontaneously improved.

In this study, we observed several AD patients at skeletal maturity with no history of treatment for DDH. Patients with AD have been reported to exhibit an abnormal morphology of the pelvis,^10,11^ a high rate of coexisting spinal congenital anomalies, such as spina bifida occulta,^12^ and high bone mineral densities of the lumbar spine, ultradistal radius, and calcaneus.^13^ As previously mentioned, primary OA of the hip is an extremely rare condition in Japan.^6^ Japanese patients with OA have been reported to have higher rates of dysplasia than American patients (46% vs 4.5%)^7^ and significantly worse dysplasia than British populations (mean CE angle 37° vs 31°).^9^ These reports and our data suggest that one-third of patients with AD in Japan have residual AD affected by treatment of DDH, and the remaining two-third patients came from a genetic background characteristic of Japanese individuals from that of Caucasians.

Some previous studies described a natural course of osteoarthritis of the hip in patients with AD.^20,21^ Hasegawa et al^20^ evaluated 86 hips in 59 patients with pre- or early stage osteoarthritis (average age 29.9 years) and 31 hips (66%) with more progressive disease (average age 7.8 years). Small values for CE angle and AHI and large values for acetabular roof angle were observed in the hips of patients with pre-osteoarthritis, which progressed to an advanced stage. However, no differences in these radiographic parameters were observed in the hips of patients in early stages of osteoarthritis. Hisatome et al^21^ reported that 7 of 61 (11%) hips (average age 38.2 years) with pre- or early stage osteoarthritis progressed to advanced stages within an average of 10.1 years. No differences in radiographic parameters were observed between the hips with maintained and progressive disease. Neither of these previous studies^20,21^ analyzed existing treatment for DDH in infancy. In this study, the average age of patients initially examined for hip pain was 40.7 years, and lower ages were observed in the DDH group (36.2 years) compared with the non-DDH group (43.3 years). In addition, a significant difference was still observed after adjustment for radiographic parameters of AD. Complications of dislocated hip joints in infancy (elongated ligamentum teres, everted labrum, and a stretched hip capsule) may accelerate development of pain in patients with DDH at skeletal maturity, prompting them to seek care for the pain earlier.

There were significant differences between the mild and severe dysplasia groups, and an increasing trend in the percentage of patients with DDH associated with severity of AD classified using acetabular angle and acetabular roof angle; however, no differences were observed in cases of severity of AD classified on the basis of CE angle and AHI. CE angle and AHI evaluations represent the position of the femoral head to the acetabulum. In contrast, acetabular angle and acetabular roof angle evaluation represents the whole structure and slope of the weight-bearing area of the acetabulum (Figure 1). The labrum of a non-dislocated hip is a thin fibrocartilaginous rim around the periphery of the acetabular cartilage. This vital cartilaginous acetabular analogue is essential for normal growth and development of the acetabulum. At the fibrocartilage–hyaline junction of the labrum and acetabulum, there may be eversional or inversional hypertrophic changes (neolimbus) in the dislocated hip.^1^ Neolimbus, which was formed during dislocation of the hip, may affect acetabulum growth even after reduction.

In conclusion, we observed that 64% patients with AD at skeletal maturity have no history of treatment for DDH. AD patients with a history of treatment for DDH have hip joint pain at a younger age and severe dysplasia, particularly when evaluated by acetabular angle and acetabular roof angle. Our data suggests that AD in patients without a history of DDH has different characteristics from AD in patients with a history of DDH.
